# Direct Interaction between Calmodulin and the Grb7 RA-PH Domain

**DOI:** 10.3390/ijms21041336

**Published:** 2020-02-17

**Authors:** Gabrielle M. Watson, Jacqueline A. Wilce

**Affiliations:** Department of Biochemistry and Molecular Biology, Biomedicine Discovery Institute, Monash University, Wellington Road, Clayton VIC 3800, Australia; gabrielle.watson@monash.edu

**Keywords:** Grb7, Calmodulin, RA-PH domain, SH2 domain, SPR

## Abstract

Grb7 is a signalling adapter protein that engages activated receptor tyrosine kinases at cellular membranes to effect downstream pathways of cell migration, proliferation and survival. Grb7’s cellular location was shown to be regulated by the small calcium binding protein calmodulin (CaM). While evidence for a Grb7/CaM interaction is compelling, a direct interaction between CaM and purified Grb7 has not been demonstrated and quantitated. In this study we sought to determine this, and prepared pure full-length Grb7, as well as its RA-PH and SH2 subdomains, and tested for CaM binding using surface plasmon resonance. We report a direct interaction between full-length Grb7 and CaM that occurs in a calcium dependent manner. While no binding was observed to the SH2 domain alone, we observed a high micromolar affinity interaction between the Grb7 RA-PH domain and CaM, suggesting that the Grb7/CaM interaction is mediated through this region of Grb7. Together, our data support the model of a CaM interaction with Grb7 via its RA-PH domain.

## 1. Introduction

**Figure 1:**
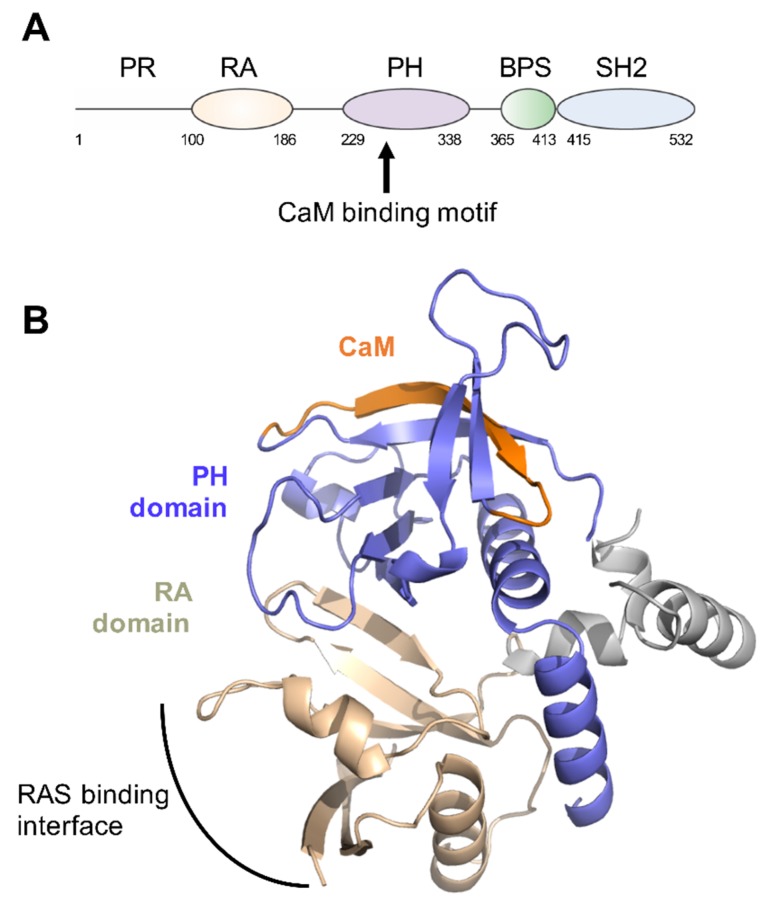
Grb7 domain structure. (**A**) Schematic depicting the arrangement of Grb7 domains and highlighting the position of the postulated calmodulin (CaM) binding site; (**B**) Model of the Grb7 RA-PH domains based upon the Grb10 RA-PH structure (PDB:3HK0). The RA domain is coloured wheat, the PH domain is purple and the residues that correspond to the Grb7 CaM-BD are coloured orange.

Growth receptor bound 7 (Grb7) protein is a cytoplasmic adapter protein that couples activated tyrosine kinase receptors to a nexus of downstream proliferative, migratory and survival signalling pathways [1,2]. Grb7 is a member of the Grb7 family of proteins that also includes Grb10 and Grb14. The three members share a conserved domain architecture comprising an N-terminal proline rich (PR) domain, followed by a central region that shares homology with *Caenorhabditis elegans* Mig-10 (the Grb and Mig region, GM) and a C-terminal Src-homology 2 (SH2) domain [3]. The GM domain, in turn, is made up of Ras-associating (RA) and Pleckstrin homology (PH) domains and a BPS (between PH and SH2) domain (Figure 1A). It is through the C-terminal SH2 domain that Grb7 is able to interact with phosphorylated tyrosines of activated upstream tyrosine kinase partners, resulting in Grb7 phosphorylation at the GM region, and propagation of downstream events. However, the other domains of Grb7 are also involved in mediating signalling outcomes. For example, the RA domain can influence proliferative signalling pathways by interacting with activated GTP bound Ras [4], the N-terminal PR domain has been reported to interact with the RNA-binding protein HuR, facilitating recruitment to stress granules [5,6], and the PH domain facilitates interactions with the cell membrane where SH2 domain mediated interactions with membrane bound receptors are formed [7].

The PH domain was also reported to bind the small, ubiquitously expressed protein calmodulin (CaM) in a calcium dependent manner [8]. The Villalobo group showed pull-down of Grb7 from cells by CaM-affinity chromatography and interactions with Grb7 from cell extract were supported by biotin-CaM detection. The interaction was further shown to regulate both Grb7’s ability to localize to membranes, and its trafficking to the nucleus [8,9,10]. CaM undergoes a conformational change upon binding calcium, allowing newly exposed hydrophobic residues to bind an array of cytosolic target proteins, including partners that are involved with regulating cell shape and migration [11,12]. For Grb7, the CaM binding site was mapped to the proximal region of the PH domain (Grb7 residues 243–256). A peptide representing this region was shown to have high affinity for CaM [13].

Together, these experiments show compelling evidence for a Grb7/CaM interaction. However, a direct Grb7/CaM interaction has never been verified with pure full-length Grb7 protein nor quantitated. Furthermore, it has been established that Grb7 can be phosphorylated on the central GM region, specifically Y188 and Y338, and this phosphorylation is required for ErbB2 mediated signalling via Grb7 [14,15]. Whether or not RA-PH phosphorylation, or additional Grb7 post-translational modifications, are also required for the Grb7/CaM interaction has not yet been explored. Lastly, while the structure of the Grb7 PH domain has not been determined, by structural homology to the Grb10 RA-PH domain (56% sequence identity) the predicted CaM binding motif corresponds to a region of β-strand (amino acid sequence: RKLWKRFFCFLRRS) (Figure 1B). This was unexpected, as it was originally postulated that the Grb7 CaM binding motif represented an α-helical target [8], and suggests a non-canonical mode of interaction.

The current study was therefore undertaken to determine whether direct interactions between CaM and purified Grb7 could be detected in vitro and in the absence of post-translational modifications or additional cellular factors. To do this we expressed and purified recombinant CaM and full-length Grb7 from *Escherichia coli* and analyzed their interaction using surface plasmon resonance (SPR) that detects molecular interactions with high sensitivity. We also produced the RA-PH domain of Grb7 in isolation, as well as the SH2 alone, in order to determine the requirement of the RA-PH domain for the Grb7/CaM interaction. We demonstrated that CaM is able to interact with full-length Grb7 in a calcium dependent manner, and that this interaction is not mediated through the SH2 domain. In contrast, we observed high micromolar affinity binding between the Grb7 RA-PH domain and CaM that is also dependent on the presence of calcium. Thus, we are able to confirm that Grb7 and CaM do indeed directly interact, although whether or not additional factors are required to augment the interaction in vivo is still open for investigation.

## 2. Results

**Figure 2:**
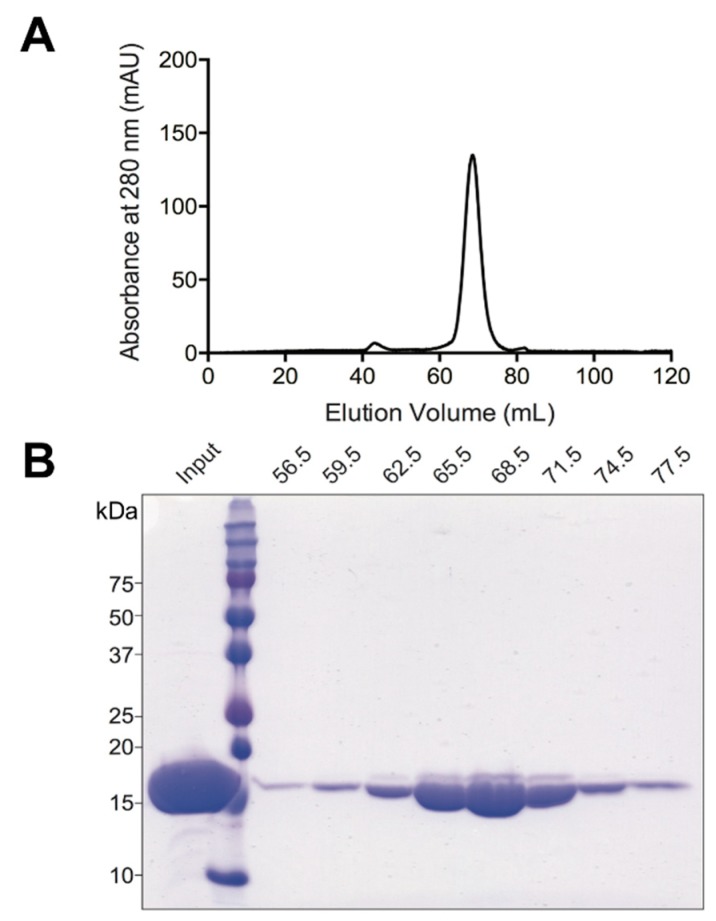
Purification of CaM. (**A**) Size exclusion chromatography of CaM: chromatogram of the size exclusion chromatography profile with absorbance at 280 nm displayed; (**B**) 15% SDS-PAGE of the resultant fractions showing purity of final CaM compared to input.

In order to verify a direct interaction between CaM and Grb7 in vitro, high purity CaM and Grb7 protein were prepared. Recombinant CaM was overexpressed in *Escherichia coli* using a previously established expression and purification protocol [16]. Figure 2 shows the quality of the protein in the size exclusion chromatography step, and its final 95% purity shown by SDS-PAGE.

**Figure 3:**
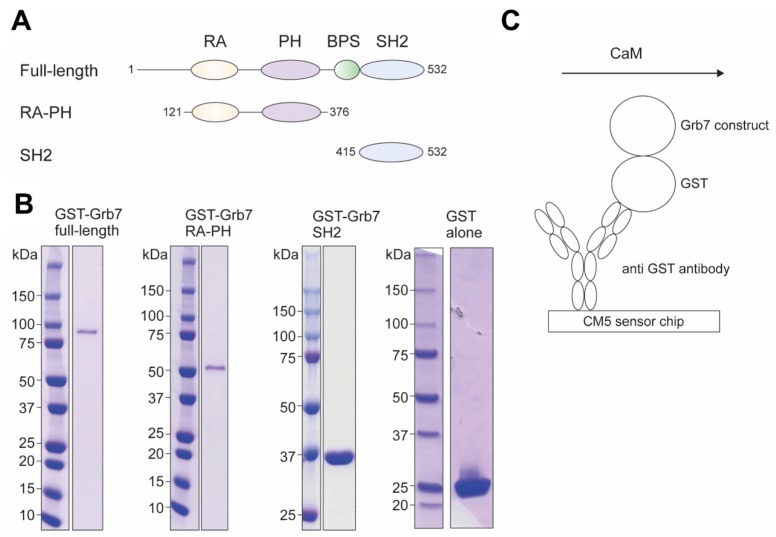
Preparation of Grb7 constructs. (**A**) The domain boundaries of Grb7 full-length, Grb7-RA-PH and Grb7-SH2 used for the preparation of GST-tagged proteins are shown. (**B**) SDS-PAGE analysis of the purified GST Grb7 constructs used for SPR experiments. (**C**) Schematic depicting the SPR experimental design. The GST Grb7 construct is captured on the surface of the biosensor chips via anti GST antibody. CaM is flowed across the surface of the chip under controlled buffer conditions, enabling interactions to be detected via SPR.

Full-length Grb7, Grb7-RA-PH and Grb7-SH2 were prepared as GST-tagged constructs to enable efficient capture on biosensor chips via anti GST-antibodies (Figure 3A). GST-tagged full-length Grb7 was overexpressed in *Escherichia coli* and purified as previously described, generating protein to > 95% purity (Figure 3B) [17]. To determine the requirement of the RA-PH region of Grb7, a GST-tagged construct that spanned the central RA and PH domains of Grb7 (residues 98–353) was cloned from the full-length Grb7. The boundaries for this construct were designed based on the boundaries reported for the Grb10 RA-PH construct for which the X-ray crystal structure was determined [18]. In the case of the Grb10 RA-PH construct, four cysteine to serine point mutations were introduced to prevent aggregation. In light of this, two corresponding point mutations (C113S and C207S) that were predicted to be distal to the predicted CaM binding interface were introduced to the Grb7 RA-PH construct. The GST Grb7 RA-PH was overexpressed in *Escherichia coli* and purified similarly to GST-tagged Grb7 full-length, generating protein to > 95% purity (Figure 3B). Finally, GST-tagged Grb7-SH2 domain was prepared as previously described [19] to produce highly pure protein as shown in Figure 3B.

The purified GST-tagged Grb7 constructs were immobilised on a CM5 sensor chip surface using GST antibody capture, as previously described for Grb7-SH2 domain [20] (Figure 3C) and purified GST was immobilized on the reference flow cell as a negative control. CaM was flowed across the sensor chip surface at a range of concentrations in buffers containing either 5 mM CaCl_2_ or 5 mM EGTA (to ensure calcium free CaM). Multi-cycle kinetics was utilized to allow a regeneration step with a buffer containing 5 mM EGTA to disrupt the Grb7/CaM interaction. All sensorgrams were double referenced by subtracting the GST alone from reference flow cell as well as buffer only injections.

**Figure 4:**
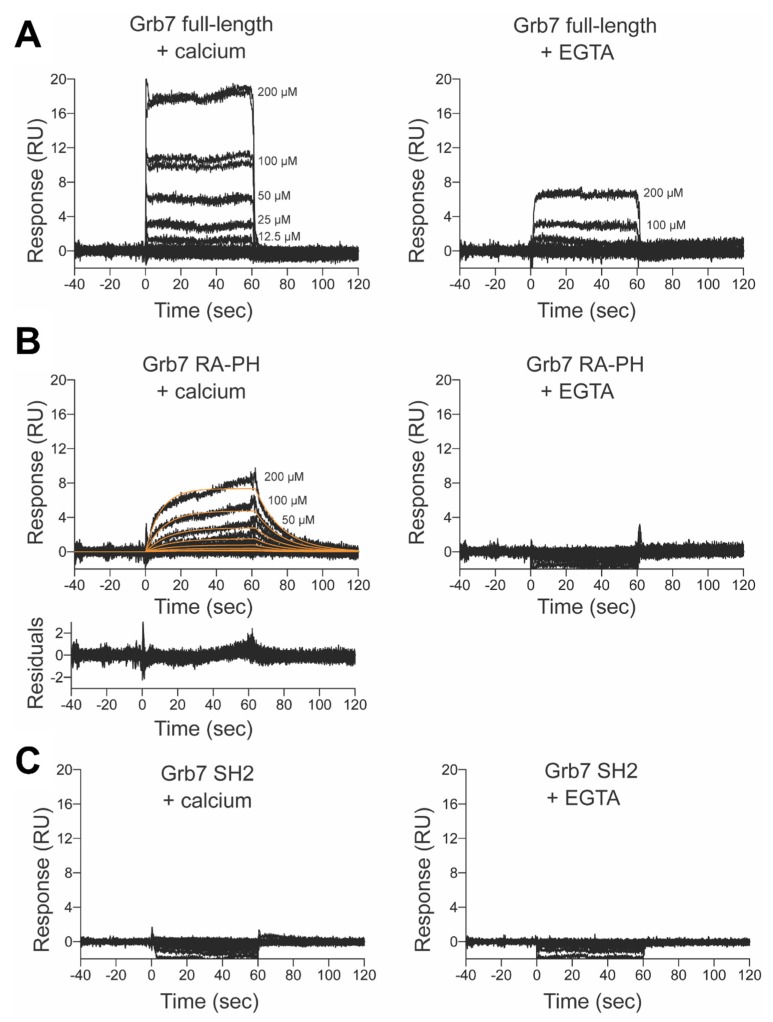
Investigating the interaction between Grb7 and CaM. Sensorgrams of CaM binding to various Grb7 domains in buffer containing 5 mM CaCl_2_ (left hand panel) or 5 mM EGTA (right hand panel). Duplicate sensorgrams are shown for each CaM concentration, as indicated. (**A**) Binding to full-length Grb7; (**B**) Binding to Grb7 RA-PH domain, with the fit to a 1:1 binding model shown in orange. The residuals of the fit are displayed in the bottom graph; (**C**) Binding to the Grb7-SH2.

In the presence of calcium we observed a direct interaction between full-length Grb7 and CaM (Figure 4A) and the observed response was approximately a third in the presence of EGTA, consistent with the Grb7/CaM interaction occurring via a calcium-triggered conformational change. The interaction occurred with fast on- and off-rates, which is typical of low affinity binding. While the range of CaM concentrations tested was not sufficient for a precise measurement of the affinity of the interaction, our data suggest a direct but low affinity interaction between recombinant CaM and full-length Grb7.

A calcium dependent interaction was also observed for CaM and the Grb7-RA-PH domain construct, with binding observed in the presence of calcium and no binding observed in the presence of EGTA (Figure 4B). The shape of the sensorgram for the Grb7 RA-PH/CaM interaction was also altered compared to that of the full-length Grb7/CaM interaction (Figure 4A). In the case of the Grb7 RA-PH construct, the dissociation occurred more slowly, allowing the dissociation and association phases of the sensorgrams to be fitted, with a calculated on-rate (*k*_a_) of 275 M^−1^s^−1^ and off-rate (*k*_d_) of 0.066 s^−1^. The slowed dissociation phase of the Grb7 RA-PH/CaM interaction suggests a higher affinity interaction compared with the full-length Grb7/CaM interaction. The determined on-rate and off-rates also permitted the direct determination of an affinity of the Grb7 RA-PH/CaM interaction with a *K*_D_ of 240 μM. This micromolar affinity is consistent with the CaM interaction with Grb7 occurring through the RA-PH domain. These data are summarised in Table 1.

Finally, no interaction between CaM and the Grb7-SH2 domain was observed in either the presence or absence of calcium (Figure 4C), supporting the Grb7/CaM interaction being mediated through the RA-PH unit. This Grb7-SH2 domain construct has been used in many previous experiments testing peptide interactions [17,21,22] and thus is a reliable negative control for demonstrating that the CaM interaction with Grb7 does not take place through the SH2 domain.

It should be noted that there was a small amount of non-specific binding detected on the GST control reference flow cell when CaM was injected at high concentrations. This subtraction of non-specific binding is evident in the Grb7-SH2 sensorgrams with negative values detected upon injection of the analyte. Nevertheless, it can be concluded that CaM binds to Grb7 via the RA-PH region in a calcium dependent manner.

## 3. Discussion

In this study, we investigated the relationship between Grb7 and the calcium sensor protein CaM. Using SPR we demonstrated that CaM binds to full-length Grb7 and that this interaction is calcium dependent. To further characterize the interaction, a construct that spans the RA-PH unit of Grb7 (and includes the postulated CaM binding motif) was cloned, expressed and purified. Using the same SPR experimental approach, we demonstrated that CaM does indeed bind to this RA-PH region of Grb7, also in a calcium dependent manner. Furthermore, no binding was detected to the Grb7 SH2 domain, supporting a RA-PH mediated interaction. This was important to ascertain as it was previously demonstrated that both apo CaM and calcium bound CaM were able to bind to the SH2 domain of Src [23].

The finding of a calcium dependent direct interaction between CaM and Grb7 is consistent with reports by the Villalobo group [8,9,10]. We identified that the Grb7/CaM interaction occurs with micromolar affinity under the conditions utilised for these in vitro experiments, which include a higher than physiological calcium concentration to ensure saturation of CaM. This is a very low affinity, considering the estimated concentration of free CaM in cells is between 50–75 μM [24]. Typically, calcium activated CaM binds to its target proteins with affinities in the range of 100 nM to 10 pm [25]. Futhermore, the Grb7 peptide, representing the CaM binding site out of the context of surrounding secondary and tertiary structure, was reported to have an affinity for CaM of 2.5 nM [13]. With the affinity measurement observed here > 1000-fold weaker than other reported interactions, it is postulated that additional cellular factors, such as post-translational modifications, and/or conformational rearrangements are likely to play a role to enhance the affinity of the Grb7/CaM interaction. Such factors have already been shown to affect Grb7 interactions with another binding partner—FHL2 [26]. In this study, phosphorylation of the Grb7-PH and SH2 domains was shown to impact the interaction—either directly or via a conformational rearrangement.

The structure of the RA-PH domain of Grb7 is not yet known; however, the Grb10 RA-PH unit was solved using X-ray crystallography and is likely a homologous structure to that of Grb7 RA-PH domain [18]. In this structure, the predicted CaM binding interface forms part of a β-strand (Figure 1B), suggesting that CaM interacts at a non-canonical site, and not an α-helical target as originally postulated [8]. Since the original report of CaM binding to the Grb7-RA-PH domain, other studies have shed light on a similar interaction—that of the interaction of CaM with the PH domain of the signalling protein Akt, though in this case the affinity of interaction was much higher (*K*_D_ = 100 nM) [27]. In this study the CaM binding interface of the Akt-PH domain was mapped using an NMR titration method, revealing a surface of interaction adjacent to the equivalent surface proposed for the Grb7-PH domain, supporting Grb7 residues 243–256 as the CaM binding motif. Whether CaM interactions are prevalent for the regulation of other PH domain-containing proteins is yet to be shown.

Although the affinities of interaction in this study are estimates, it is of interest to compare the interactions of CaM with full-length Grb7 compared with Grb7-RA-PH domain. It is particularly noticeable that the sensorgram shapes greatly differ between binding of CaM to full-length Grb7 and binding to the Grb7 RA-PH domain. The slower off-rate seen for the Grb-RA-PH domain reflects a higher affinity interaction compared to that of the full-length Grb7. Such an occurrence could be due to a difference in the available Grb7 binding surface between the two constructs. It was previously postulated by the Lyons group that Grb7 is regulated through an intra-molecular association whereby the SH2 domain interacts with the RA-PH domain. This interaction was demonstrated by measuring chemical shift changes in ^15^N-^1^H-HSQC spectra of the Grb7-SH2 domain upon titration with the Grb7-RA-PH domain [26]. Such an interaction is entirely consistent with our findings. The CaM binding site may be partly occluded in the case of full-length Grb7 due to the presence of the SH2 domain, and not in the case of the Grb7-RA-PH alone. Accordingly, our findings also support the proposal of a Grb7 structural rearrangement that exposes the RA-PH domain upon CaM binding. CaM binding could therefore open up the Grb7 structure, facilitating RA-PH interactions with their binding partners.

Finally, the current study demonstrates a low affinity interaction between CaM and the Grb7 RA-PH domain. Due to the high level of homology between Grb7 and family members Grb10 and Grb14, it could be predicted that CaM may also interact with these proteins in a similar manner. Indeed, initial work has revealed that Grb10 and Grb14 are also bound by CaM and that the interaction site may be similar to that proposed for Grb7 [13]. Whether these interactions occur via direct interaction or with significant affinity, and the cellular implications, awaits further investigation.

## 4. Materials and Methods

### 4.1. Protein Expression and Purification

Glutathione-S-transferase (GST) tagged Grb7 SH2 domain (encoding residues 415–532) and a GST alone control were expressed and purified as previously described [17]. Grb7 full-length (encoding residues 1–532), kindly provided by Peter Leedman, was subcloned into the pGex6p2 expression vector and expressed as a soluble GST tagged protein in the *Escherichia coli* BL21 (DE3) pLysS cell line. The cells were lysed by sonication in buffer comprising PBS (pH 7.4), 2 mM EDTA, 0.5% (v/v) Triton X-100 and 1 mM dithiothreitol (DTT), before the Triton X-100 concentration was increased to 1% (v/v) and stirred for 1 h at 4 °C. The cleared lysate was subsequently purified by glutathione affinity chromatography (including a 2 M NaCl wash step to remove bound nucleic acid) and size exclusion chromatography (S200 16/60, GE Healthcare) in a buffer comprising 50 mM NaPO_4_^3−^ (pH 7.0), 300 mM NaCl, 10% (v/v) glycerol and 5 mM DTT.

The Grb7 RA-PH construct (encoding residues 98–353) was cloned into the pGex6p2 plasmid, and two point-mutations introduced (C113S and C207S) using QuikChange site-directed mutagenesis [28] to prevent unwanted oligomerisation, based on a Grb10 RA-PH construct [18]. The validity of the construct and the introduction of the desired point mutations were verified using the Micromon (Monash University, Melbourne, Australia) sequencing service. GST tagged Grb7 RA-PH C113S/C207S (herein referred to as GST-Grb7 RA-PH) was expressed in the *Escherichia coli* BL21 (DE3) plysS Rosetta2 cell line, and purified as per Grb7 full-length, with the exclusion of the 2 M NaCl wash during the glutathione affinity chromatography step.

The pET14b plasmid containing CaM was kindly provided by Antonio Villalobo, and expressed in the *Escherichia coli* BL21 (DE3) pLysS cell line. Similar to previously described [16], in a buffer comprising 50 mM Tris-HCl (pH 7.5), 2 mM EDTA and 0.2 mM PMSF, cells were lysed by sonication and, following the addition of 5 mM CaCl_2_, the soluble fraction purified by hydrophobic interaction chromatography (Hitrap Phenyl HP, GE Healthcare, Parramatta, Australia). The bound CaM was eluted in a buffer comprising 50 mM Tris-HCl (pH 7.5), 1 mM EGTA and further purified by size exclusion chromatography (S75 16/60, GE Healthcare, Parramatta, Australia) in 50 mM Tris-HCl (pH 7.4), 150 mM NaCl, 5 mM CaCl_2_. For binding experiments, CaM was dialysed overnight into the appropriate analyte running below (see below).

### 4.2. Surface Plasmon Resonance (SPR)

SPR experiments were performed on a BIAcore T100 at 25 °C similar to previously described [17,20,21,22]. In a buffer comprising 50 mM NaPO_4_^3−^ (pH 7.4), 150 mM NaCl and 1 mM DTT, polyclonal anti-GST antibody (Abcam, Cambridge, UK) was immobilised on a BIAcore CM5 series S sensor chip (GE Life Sciences, Parramatta, Australia) via amine coupling with bound responses between 934 and 1081 response units (RU). GST-alone was immobilised on the reference flow-cell, and GST-Grb7-SH2, GST-Grb7 RA-PH and GST-Grb7 full-length immobilised on the active flow-cells with capture levels of 212 RU, 266 RU, 391 RU and 368 RU, respectively. In duplicates, at 30 μL/min, CaM at various concentrations (1.56–200 μM) was injected over the chip surface for 60 s, followed by a dissociation time of 180–360 s. The running buffer comprised 50 mM Tris (pH 7.4), 150 mM NaCl, 1 mM DTT, and either 5 mM CaCl_2_ or 5 mM ethylene glycol-bis(β-aminoethyl ether)-N,N,N′,N′-tetraacetic acid (EGTA). When the running buffer contained CaCl_2_, a regeneration step was incorporated comprised of an 80 s injection of running buffer including 5 mM EGTA. The responses on the active flow cells were double referenced by subtracting the GST-alone reference flow cell as well as buffer only injections. The data were analysed using Scrubber2.0 (BioLogic Software, Campbell, Australia).

## Figures and Tables

**Table 1:** Binding parameters of the calcium induced interaction between CaM and various Grb7 domains.

Binding Parameter ^#^	RA-PH	Full-Length	SH2
*k*_a_ (M^−1^s^−1^)	275 ± 2	N/A	N/A
*k*_d_ (s^−1^)	0.0660 ± 0.0003	N/A	N/A
Rmax (RU)	16.2 ± 0.1	ND	ND
Theoretical Rmax (RU)	120.8	72.7	115.9
*K*_D_ (μM)	240 ± 2	ND	ND
Residuals squared	0.384	N/A	N/A

^#^ Binding parameters calculated from fits to a single site binding model. The errors shown are the error arising from the fits. CaM was injected at 8 concentrations with each concentration tested in duplicate. Experiments were performed in a running buffer containing 50 mM Tris-HCl (pH 7.4), 50 mM NaCl, 5 mM CaCl_2_ and 1 mM DTT. N/A = not applicable. ND = not determined.

## Abbreviations

Grb7 GST	growth receptor bound protein 7 glutathione-S-transferase
CaM	calmodulin
DTT	dithiothreitol
EGTA	ethylene glycol-bis(β-aminoethyl ether)-N,N,N′,N′-tetraacetic acid
SH2	Src-homology 2
SPR	surface plasmon resonance
RU	response units
